# Metabolic labeling of plant cell cultures with K^15^NO_3 _as a tool for quantitative analysis of proteins and metabolites

**DOI:** 10.1186/1746-4811-2-14

**Published:** 2006-09-04

**Authors:** Wolfgang R Engelsberger, Alexander Erban, Joachim Kopka, Waltraud X Schulze

**Affiliations:** 1 Max-Planck Institut für Molekulare Pflanzenphysiologie, Am Mühlenberg 1 14476, Golm, Germany

## Abstract

Strategies for robust quantitative comparison between different biological samples are of high importance in experiments that address biological questions beyond the establishment of protein lists. Here, we propose the use of ^15^N-KNO_3 _as the only nitrogen source in Arabidopsis cell cultures in order to achieve a metabolically fully labeled cell population. Proteins from such metabolically labeled culture are distinguishable from unlabeled protein populations by a characteristic mass shift that depends on the amino acid composition of the tryptic peptide analyzed. In addition, the metabolically labeled cell extracts are also suitable for comparative quantitative analysis of nitrogen-containing cellular metabolic complement. Protein extracts from unlabeled and from standardized ^15^N-labeled cells were combined into one sample for joined analytical processing. This has the advantage of (i) reduced experimental variability and (ii) immediate relative quantitation at the level of single extracted peptide and metabolite spectra. Together ease and accuracy of relative quantitation for profiling experiments is substantially improved. The metabolic labeling strategy has been validated by mixtures of protein extracts and metabolite extracts from the same cell cultures in known ratios of labeled to unlabeled extracts (1:1, 1:4, and 4:1). We conclude that saturating metabolic ^15^N-labeling provides a robust and affordable integrative strategy to answer questions in quantitative proteomics and nitrogen focused metabolomics.

## Background

Plants adjust to developmental and environmental variability with respective changes in protein abundance and enzyme activity. Thus, protein expression and metabolite pools in cells can be highly dynamic and the abundance and activity of specific proteins can vary greatly during growth and development or in response to biotic and abiotic stress. Therefore, it is of great biological interest to be able to quantitatively compare the subproteomes of different developmental stages or to quantitatively examine the dependent or independent responses of metabolic or signaling pathways under specific conditions. Thus combined analysis of metabolites and proteins may yield novel information about regulatory processes in plants [1].

Liquid-chromatography coupled mass spectrometry has in the recent years become a widely applied tool in quantitative proteomic approaches also in plant sciences [2-4]. Mass spectrometry is especially powerful to identify and quantitate changes in post-translational modifications, which often play important roles in protein function and regulation [5,6]. However, quantitative comparisons between independent samples remain a challenging task. Label-free approaches termed 'protein correlation profiling' rely on the relative quantitation of ion intensities between independent LC-MS/MS runs require accurate reproducibility of retention times in combination with elaborate data analysis [7,8]. Other label-free techniques use the number or recorded spectra for each protein as a comparative measure [9,10].

Due to difficulties in peak assignments between independent LC-runs of complex mixtures, many quantitative proteomic strategies rely on the incorporation of stable isotopes into proteins or peptides, which are then quantitatively compared to an unlabeled control sample based on their mass spectra [11]. Relative protein abundance can be calculated from the intensities of the labeled versus the unlabeled labeled forms of the same tryptic peptides in the same mass spectrum (for review see [12,13]). Stable isotope labeling of protein samples can in principle be achieved by chemical modification of proteins and tryptic peptides. Initially, ^18^O labeling was used to differentially label peptides of protein samples during the digest with trypsin [14]. This approach is still used today for relative comparison between two samples [4,15]. Later, isotope coded affinity reactive tags were developed which tag specific amino acid species and allow for specific enrichment of the labeled tryptic peptides [16]. This variety of tags has successfully been used in an number of comparative proteomic studies involving comparisons of changes in protein abundances [17-19] or characterizing changes in posttranslational modifications [20,21]. Furthermore, stable isotope labeled synthetic peptides are being used as internal standard peptides for a variety of targeted assays involving quantitative mass spectrometry [22,23]. However, the drawbacks of chemical labeling strategies are that only a rather small subset of tryptic peptides are being tagged and that experimental variability may be introduced during the labeling processes.

The first studies making use of metabolic labeling for protein quantitation by mass spectrometry relied on growth of bacterial or yeast cells on ^15^N-enriched medium [24,25]. However, since the mass shift introduced by ^15^N labeling depends on the amino acid composition of each tryptic peptide, knowledge of the peptide sequence is necessary to calculate the expected mass difference to the labeled or unlabeled partner. Thus, full metabolic labeling has recently been widely replaced by labeling of only specific amino acids (SILAC), such as lysine, arginine or leucine to introduce a fixed mass shift between labeled and unlabeled peptide pairs [26-28]. SILAC works especially well with mammalian cell cultures to which essential amino acids can easily be supplied in a stable isotope labeled form and full incorporation into the proteome of the cells is ensured. In yeast, auxotrophic mutants inhibited in the synthesis of arginine or lysine have been used for full incorporation of stable isotope labeled amino acids. The SILAC approach has successfully been applied to quantitative proteomic studies of the formation of signal-dependent protein complexes [29], in modification-dependent protein-protein interaction screens [30,31], and to analyses of the dynamics of signal-dependent phosphorylation events [32-34]. In plants, labeling of arginine, lysine or leucine has also been achieved using cell cultures [35], and an average of 70% to 80% incorporation of the metabolic label was obtained. Possibly, by using auxotrophic mutants in certain amino acid synthesis pathways, higher degrees of labeling may be feasible. In general it has to be considered that SILAC labeling is a costly exercise, especially if high concentrations of amino acids need to be supplied, such as for plant cell cultures.

Therefore, in plant biology, quantitative proteomic approaches so far mainly relied on chemical modification of proteins to study membrane lipid rafts [36], on ^18^O labeling in tryptic digests, e.g. to characterize purity of plant plasma membrane purifications [4], or on label-free quantitation by spectrum count as comparison between independent samples [2]. More recently, the iTRAQ reagent can be used in studies comparing more than two samples [37]. However, metabolic labeling strategies of whole proteomes have the advantage over chemical labeling that samples to be compared can be processed together through the complete analytical process from extraction of combined biological samples, isolation of metabolic or proteomic complements to quantitative analysis. Since biochemical techniques, such as the isolation of proteins, their fractionation and enrichment introduce much more heterogeneity than what is known from analyses of mRNA, reduction of sample variability is of great importance [13]. Also for metabolites, comparative quantitation to date relies on the analysis of a large number of replica samples [38]. Only recently has the use of ^13^C-labeling been proposed as a strategy for quantitative analysis of metabolite profiles [39,40]. In addition N labeling in plants has been successfully applied to flux studies and tracer analysis indicating the feasibility of generating saturated N labeled metabolomes [41,42].

In complementation to metabolic N-flux analysis, we propose the use of full ^15^N-labeling of plant cell cultures in comparative quantitative proteomics and metabolomic approaches as a cheaper alternative to SILAC labeling and a more robust and accurate method for quantitation compared to label-free protein correlation profiling. For the reasons noted above, ^15^N-labeling of intact plant has yet only been applied to analysis of proteins by NMR [43] or metabolic flux studies [41,42], which do not require full incorporation of the label. In addition, the approach described here is the first example of a labeling strategy that can be robustly applied to the protein and metabolite complement of the same sample.

## Results and discussion

Since first proteomic experiments involving metabolic labeling with ^15^N involved growth of yeast cells in ^14^N and ^15^N medium for the quantitation of phosphorylation [24], the technique of ^15^N-labeling for proteomic research had mostly remained in the field of microbiology. Exceptions are studies using ^15^N-labeled microorganisms as a food source for metabolically labeling Drosophila [44] and ^15^N-labeling of rats by the use of ^15^N-labeled algal food [45]. In this proof-of-principle study, we use ^15^N-labeling to quantify protein and metabolite abundance in known mixtures of labeled and unlabeled cells.

### Quantitative analysis of known mixtures of protein extracts

Metabolic ^15^N-labeling introduces the stable isotope in each nitrogen atom in the labeled organism. Therefore, every observable peptide from mixtures of unlabeled (^14^N) and ^15^N-labeled samples should occur in pairs and thus be suitable for quantification. In a first step, labeled protein extracts were analyzed for full incorporation of ^15^N into all proteins after 14 days of continuous growth in presence of K^15^NO_3_. Comparison of protein identifications in database searches with and without ^15^N as a parameter for fixed modification (Tab. 1) showed that 98.3% of all proteins of the labeled cells were identified only in their labeled form, while for 2% of the proteins, a residual minor peak was observed also for the ^14^N form. This indicates that ^15^N incorporation into the proteome of plant cell cultures is as complete as it can be achieved with 98 atom% enriched KNO_3_. Most importantly, we observed no significant protein to protein variation for the incorporation of the label.

**Table 1:** Proteins and the corresponding peptides identified in unlabeled protein extract (^14^N extract) and ^15^N labeled extract (^15^N extract) in two consecutive database searches without (^14^N search) and with (^15^N search) ^15^N as a fixed modification.

	Proteins		Peptides	
	14N search	15N search	14N search	15N search
14N extract	195	1	536	2
15N extract	4	167	6	415

**Figure 1:**
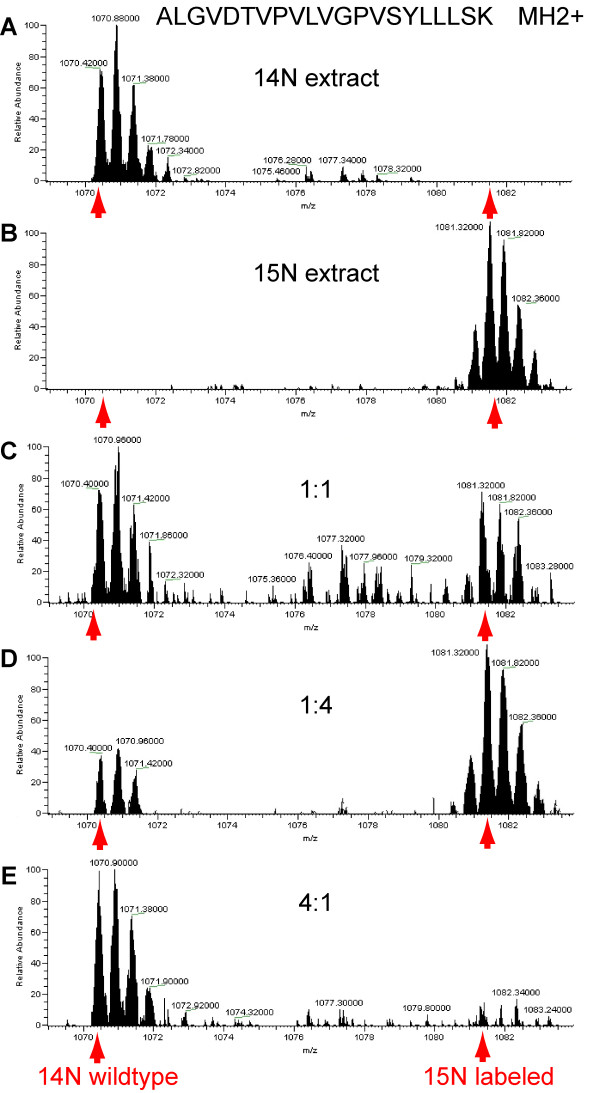
Mass spectra of the peptide ALGVDTVPVLVGPVSYLLLSK in protein extracts form unlabeled cells (A), ^15^N-labeled extracts (B), and in mixtures of unlabeled and labeled extract at a ratio of 1:1 (C), a ratio of 1:4 (D), a 4:1 ratio (E). The expected mass to charge ratios of unlabeled and labeled peptides are indicated by arrows.

In the following experiments, protein extracts of unlabeled (^14^N) cell cultures were mixed with extracts of ^15^N-labeled cell cultures at known mixing ratios of 1:1, 1:4 and 4:1. Quantitative analysis of the intensity ratios of labeled and unlabeled peptide pairs indeed corresponded to the mixing ratios (Fig. 1). Taking the doubly-charged peptide ALGVDTVPVLVGPVSYLLLSK as an example, the quantitation revealed that in unlabeled cell extracts no pairing peak was found at the expected position of the labeled peptide (Fig. 1A). In contrast, the ^15^N-labeled extract contained no visible peak at the expected position of the ^14^N-form of the respective peptide (Fig. 1B). In the 1:1 mixture of the two protein extracts, labeled and unlabeled form of the peptide were clearly identified and quantified with a ratio of 0.92 (Fig. 1C), in the 1:4 mixture the intensity ratio of labeled and unlabeled form was 2.5 (Fig. 1D), while in the 4:1 mixture the ratio was found to be 0.18 (Fig. 1E). In average in all of the above protein extract mixtures, 85% of all identified proteins were quantified, and the average intensity ratios of all quantified proteins were not significantly different (χ^2^-test) from the expected values (Tab. 2). However, it has to be kept in mind, that ratio quantitation can become limited by the signal-to-noise ratio of the mass spectrometer [26] especially if there are large differences between the two samples. In those cases, it will not be possible to accurately reproduce the precise mixing ratios in the measured ratios of the peak pairs.

**Table 2:** Overview of the intensity ratio of ^14^N and ^15^N tryptic peptide pairs and the number of proteins identified in the two database searches.

mixing ratio	mean ratio	ID in ^14^N search	ID in ^15^N search	% quantified
1:1	0.89 ± 0.2	87	53	87.4
1:4	5.69 ± 2.0	29	145	93.1
4:1	0.29 ± 0.1	149	18	75.0

There are two main reasons why proteomic quantification using full ^15^N-labeling is not as straight forward for automated high-throughput analysis. Firstly, the pairs of tryptic peptides are separated by a mass difference that depends on the number of ^15^N atoms in the respective peptide sequence. Therefore, the peptide sequence has to be determined by a database search before the mass difference to the labeled or unlabeled partner can be calculated, and this mass difference is different for each unique peptide (Tab. 3). This is actually the most critical disadvantage of the ^15^N-labeling method as it makes automated peak pair recognition impossible during data-dependent acquisition of mass spectra or in automated analysis of the raw files. Secondly, the isotopic distribution of the mass spectrum of a ^15^N-labeled peptide is slightly altered. This is so because the labeling substance, in this case KNO_3_, was used only in an isotopic enrichment of 98 atom% ^15^N. As the number of nitrogen atoms as a proportion of the total number of atoms in a peptide is rather high, the isotopic distribution of the peptide ion is influenced by the degree of enrichment of ^15^N. Similarly to previous work using full ^15^N- labeling as a method for quantification in proteomics [24,44], we do not account for this effect. However, other research groups have developed algorithms that correct for isotope enrichment effects [46] or make use of it for quantitative analysis [47]. Despite some drawbacks in the automated analysis, full ^15^N-labeling is a cheaper alternative to metabolic labeling with SILAC (Tab. 5). With the concentrations of amino acids used for SILAC labeling of plant cell cultures [35] and the concentrations of nitrate used in the labeling medium in this study, the K^15^NO_3 _labeling method is up to 10-times less expensive than SILAC labeling.

**Table 3:** A subset of tryptic peptides from glyceraldehyde-3-phosphate dehydrogenase C subunit (At3g04120) that were identified by mass spectrometry and subsequent database search (^14^N-search, see methods for details). For each peptide the expected mass difference was calculated based on the measured mass to charge ratio (MCR). Asterisks mark those peptides that have also been identified in the database search with ^15^N as fixed modification (^15^N-search, see Methods for details)

**Sequence**	**measured mass 14N**	**MCR 14N [Th]**	**Charge**	**calculated mass difference**	**calculated mass 15N**	**MCR 15N [Th]**
AASFNIIPSSTGAAK	1434.42	718.22	2	16.95	1451.37	726.69
DAPMFVVGVNEHEYK	1733.42	867.72	2	18.94	1752.36	877.18
FGIVEGLMTTVHSITATQK *	2033.69	1017.85	2	18.94	2052.63	1027.32
GILGYTEDDVVSTDFVGDNR	2170.55	1086.28	2	23.93	2194.48	1098.24
LVSWYDNEWGYSSR	1760.47	881.24	2	19.94	1780.41	891.20
SDLDIVSNASCTTNCLAPLAK	2248.71	1125.36	2	23.93	2272.64	1137.32
VPTVDVSVVDLTVR *	1497.55	749.78	2	16.95	1514.50	758.25
YDSVHGQWK	1118.39	560.20	2	13.96	1132.35	567.17

**Table 5:** Overview of the costs of SILAC labeling and full ^15^N-labeling for plant cell cultures^a^. Depending on the compounds and concentrations used in the medium, K^15^NO_3 _labeling is 7 to 12 times cheaper per liter medium.

**Compound**	**Amount in package (g)**	**Cost per package (€)**	**Cost per g (€)**	**Final concentration in medium (mM)**	**Price per L (€)**	**Cost Factor**
^13^C_6_-R	0.100	750 ^b^	7500	0.8 ^c^	972	13
^13^C_6_-K	0.100	800 ^b^	8000	0.8 ^c^	1037	13
D_3_-L	0.100	550 ^b^	5500	0.8 ^c^	590	8
K^15^NO_3_	1	75 ^b^	75	10 ^d^	77	1

^a ^for comparison, the iTRAQ Multiplex Kit costs approximately 1000 € (Applied Biosystems, Germany), allowing for 10 pairwise labelling reactions.

^b ^approximate prices according to SigmaAldrich, Germany

^c ^most efficient concentration used in Gruhler et al. 2005, MCP [35]

^d ^as used in this study

### Quantitative analysis of known mixtures of metabolic extracts

For metabolites, quantitation was based on the ion intensities of characteristic fragment ions [39]. In total, among the nitrogen-containing compounds, 14 amino acids (ala, asp, cys, glu, gly, ile, lys, met, phe, pro, thr, trp, tyr, val) and two polyamines (spermidine, putrescine) were analyzed. We estimated an average of 91% incorporation of ^15^N-label into metabolites, ranging from 70% for alanine to the maximally achievable 98% for tryptophan and putrescine.

**Figure 2:**
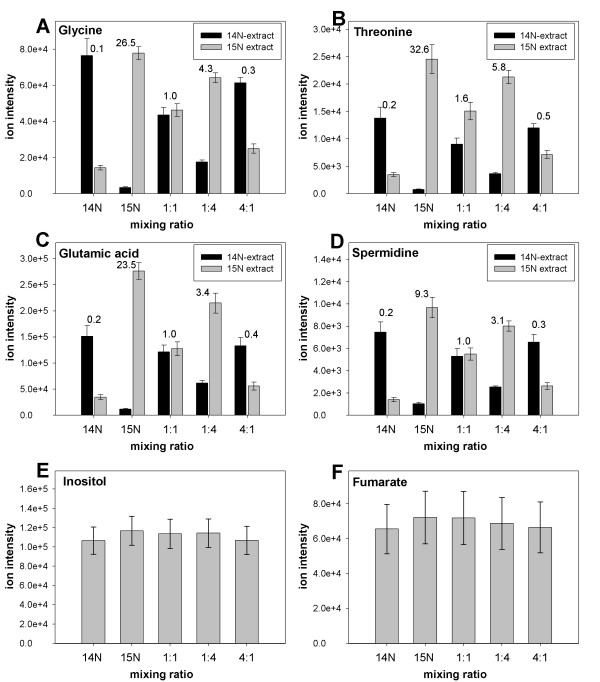
Ion intensities of characteristic fragment ions for 14N and 15N isoforms of metabolites in unlabeled cells (14N), ^15^N-labeled cells (15N) and in extracts of 1:1, 1:4, and 4:1 mixtures of unlabeled to labeled cells is shown for the amino acid glycine (A), the amino acid threonine (B), the amino acid glutamic acid (C), and the polyamine spermidine (D). Non-N-containing compounds inositol (D) and fumarate (E) were used as a control for equal extract material. Each mixture was measured in five independent samples, mean of five replica ± standard deviation are shown, and ratios between ^15^N-form and ^14^N-form for each cell mixture are shown above the bars. Average labeling efficiency of all N-containing metabolites was 91.32% as estimated from the ^15^N to ^14^N ratios in the ^15^N-labeled extract.

Mass isotopomer analysis of the saturated ^15^N-labeled cells indicated the expected portion, namely approximately 2% of residual ^14^N in all observed ^15^N-containing metabolite preparations (Fig. 2A–D, ^15^N samples). The observed mixing ratios of the unlabeled and labeled metabolite extracts show the expected tendencies. For example, ^15^N/^14^N ratios for glycine were found to be 1.0, 4.3 and 0.3 in 1:1, 1:4, and 4:1 mixtures (Fig. 2A). However, all 4:1 ratios were over-estimated without correction for the contribution of natural abundances of ^13^C (Fig. 2A–D, higher isotope peak in ^14^N-extracts). Natural abundance of nitrogen and carbon isotopes as well as discrimination effects during metabolism may account for these observations and the differences between metabolites [48,49]. In contrast to peptide analysis, we therefore conclude that when analyzing mixing ratios of compounds with one or few N-atoms the natural isotope abundance of ^13^C (1.10%) needs to be considered, whereas the contribution of natural abundance of ^15^N (0.37%) may be neglected (Fig. 2 A–D). In addition two further sources of errors were tested: (1) The two original cell cultures, labeled and unlabeled may have different metabolite pools and (2) equal combination of labeled and non-labeled samples might be inaccurate. Exemplary metabolites which do not contain nitrogen, demonstrate slight non-significant changes in pool size and negligible errors in the 1:1, 1:4, and 4:1 mixtures (Fig. 2 E–F). While glycine, inositol or fumarate levels were unchanged in the two original cell cultures (Fig. 2.A, E–F), threonine (Fig. 2B), glutamate (Fig. 2C), and to a minor extent spermidine (Fig. 2D) had accumulated in the ^15^N-labeled cells. Although the two cell cultures were grown in the same medium composition and under the exact same growth conditions, changes in metabolite contents may have resulted from slight differences in handling during harvesting (e.g. intensity of washing, speed of freezing). As a consequence, we propose that those pool-size changes in the originating cells need to be experimentally considered for the purpose profiling experiments of the N-containing metabolic complement.

Using the unmixed extracts (^14^N-extract and ^15^N-extract, Fig. 2) to estimate the contribution of natural abundance of ^13^C to higher isotope fragmentation peaks, we calculated the expected ratios of ^15^N/^14^N for all metabolites in the 1:1 mixture (Tab. 4). The calculation also takes into account that pool sizes (ion intensities) for certain metabolites were significantly different in the unmixed original cells. It becomes apparent that in most cases the measured ratios in the 1:1 mixture are rather accurate (deviation < 10%), and only for those metabolites, which display large differences in concentration in the original unmixed cells, measured ratios were over- or underestimated. Alanine was found in higher concentrations in unlabeled cells and therefore the expected mixing ratio was strongly underestimated due to signal-to-noise limitation of the ^15^N/^14^N ratios. The contrary holds true for methionine and putrescine which show higher concentrations in the ^15^N-labeled cells leading to a strong over-estimation of expected ratios. These examples underline the importance of including the unmixed extracts in the analysis. It has been described also for quantitative protein analysis that large ratios lead to larger relative errors, basically because the ratio is then defined by the signal to noise ratio of the mass spectrometer [26].

**Table 4:** Measured ratios of ^15^N/^14^N forms of metabolite fragment peaks in 1:1 mixtures of labeled and unlabeled extracts compared to their expected values. Expected ratios were calculated based on the fragment spectra ratios in the unmixed extracts (^14^N-extract, ^15^N-extract). The difference of the measured values to the expected values is expressed as percentage of the expected value.

	**measured ratios**	**expected ratios**	**difference to expected (%)**
ala	0.31	0.11	191.3
asp	7.52	7.29	3.2
cys	1.29	1.51	-14.4
glu	1.05	0.86	22.1
gly	1.04	0.93	11.9
ile	2.43	2.36	3. 0
lys	1.27	1.24	2.8
met	3.91	44.54	-91.2
phe	2.31	2.28	1.5
pro	2.54	2.49	1.9
thr	0.53	0.44	19.8
trp	2.51	2.55	-1.7
tyr	1.82	1.97	-7.8
val	1.54	1.43	7.5
put	7.16	9.48	-24.5
spe	1.03	0.97	7.0

The examples of different metabolite pool sizes (ala, met, glu, putrescine) in the ^14^N-cells compared to the ^15^N-cells actually reflect the true experimental situation, in which two cell extracts of unknown concentrations would be mixed in a 1:1 ratio, and those compound differing significantly from a 1:1 ratio of the ion intensities are concluded to be of different pool size in the original cells. The same experimental strategy generally also applies to proteins, and has already been successfully been used to detect proteins specifically interacting with a bait protein or peptide from background interaction partners or to describe temporal changes in protein abundance [29,30,32]. However, in the experiments described here, no variation between proteins was observed, since protein extracts were mixed based on total protein content, while for metabolite analysis, frozen cell material was mixed on a fresh weight basis (see Methods for details). Thus, the labeling and mixing strategy described here is well suitable for accurate detection of small changes and also ideal for screening for large deviations from an expected ratio, e.g. in presence/absence studies.

### Alternative labeling strategies

A drawback of the ^15^N-labeling strategy for metabolomic analyses can be seen in the fact that quantitative information can only be obtained for nitrogen-containing metabolites. Thus, for quantitative information of a full metabolite profile, metabolic labeling with ^13^C would be a more promising strategy, as has already been proposed in an experiment using yeast cells [39]. However, for protein analysis full ^13^C-labeling is not suitable: The high proportion of carbon atoms in tryptic peptides would lead to very large mass differences between labeled and unlabeled peptides making unambiguous detection of labeled and unlabeled ion pairs very difficult.

## Conclusion

In this study we used K^15^NO_3 _to metabolically label plant cell cultures for comparative proteomic and metabolomic experiments. We demonstrate that quantitative comparison using mixtures of labeled and unlabeled cell extracts is (i) approximately 10-times less expensive per liter of cell culture compared to SILAC labeling for plants or the iTRAQ reagent and (ii) accurate at the level of single mass spectra. Data supporting quantitative statistics are generated at the level of single mass spectra for peptides and those metabolites which comprise the N-metabolome. It is (iii) the first approach describing possibilities of a joined quantitative analysis utilizing mass isotopomer ratios of proteins and metabolites from the same cell extract. Thus, we believe that metabolic ^15^N-labeling may be applied to a wide range of biological questions in plant science which require quantitative proteomic and metabolomic analysis.

## Methods

### Metabolic labeling of cell cultures

Arabidopsis cell cultures derived from leaves [50] were grown in JPL medium [51] with 19 mM potassium nitrate as the sole nitrogen source. Potassium nitrate was supplied either in normal form or in ^15^N-enriched form (98 atom% K^15^NO_3_, Sigma-Aldrich). Cells were subcultured every 7 days using 10% of the culture volume. The remaining cells were harvested by suction over a filter plate and frozen at -80°C.

### Protein extracts and mixing

Frozen cells were ground and extracted in 50 mM TRIS-HCl pH 7.5, 1% NP-40, and a protease inhibitor mixture (Complete Tabs, Roche). Cell debris was pelleted by centrifugation and 200 μg of Protein was precipitated with TCA. Protein was resuspended in a small volume (approx. 20μL) of 6 M Urea/2 M thiourea pH8 and in-solution digested with trypsin after reduction in DTT, alkylation with iodoacetamide and dilution of the sample with 4 volumes of 50 mM NH_4_HCO_3 _was performed as described [30].

### Mass spectrometric analysis of proteins and database search

Tryptic peptide mixtures were then desalted on STAGE tips [52] before LC-MS/MS analysis using nanoflow HPLC (Proxeon Biosystems, Odense, Denmark) and a linear ion trap instrument (LTQ, Thermo Electron, San Jose, CA, USA) as mass analyzer. Peptides were eluted from the analytical column (Reprosil C18, Dr. Maisch GmbH, Tübingen, Germany) by a linear gradient running from 10 % to 50 % acetonitrile in 110 minutes and sprayed directly into the LTQ mass spectrometer. Proteins were identified by tandem mass spectrometry (MS/MS) by information-dependent acquisition of fragmentation spectra of multiple-charged peptides. Additional data-dependent fragmentation (MS3) was used to confirm identity of ambiguous proteins [53]. Fragment spectra were then searched against a non-redundant version of the Arabidopsis protein Database (TIGR6) using the Mascot algorithm (Matrix Science, UK, ). The following search parameters were applied: Peptide mass tolerance 800 ppm, MS/MS tolerance 0.8 Da, methionine oxidation and carbamidomethylation of cysteine were set as variable modifications. For each sample, two database searches were carried out, one without fixed modifications ('14N-search') and one with ^15^N-amino acids as a fixed modification ('15N-search').

### Quantitative analysis of protein

**Figure 3:**
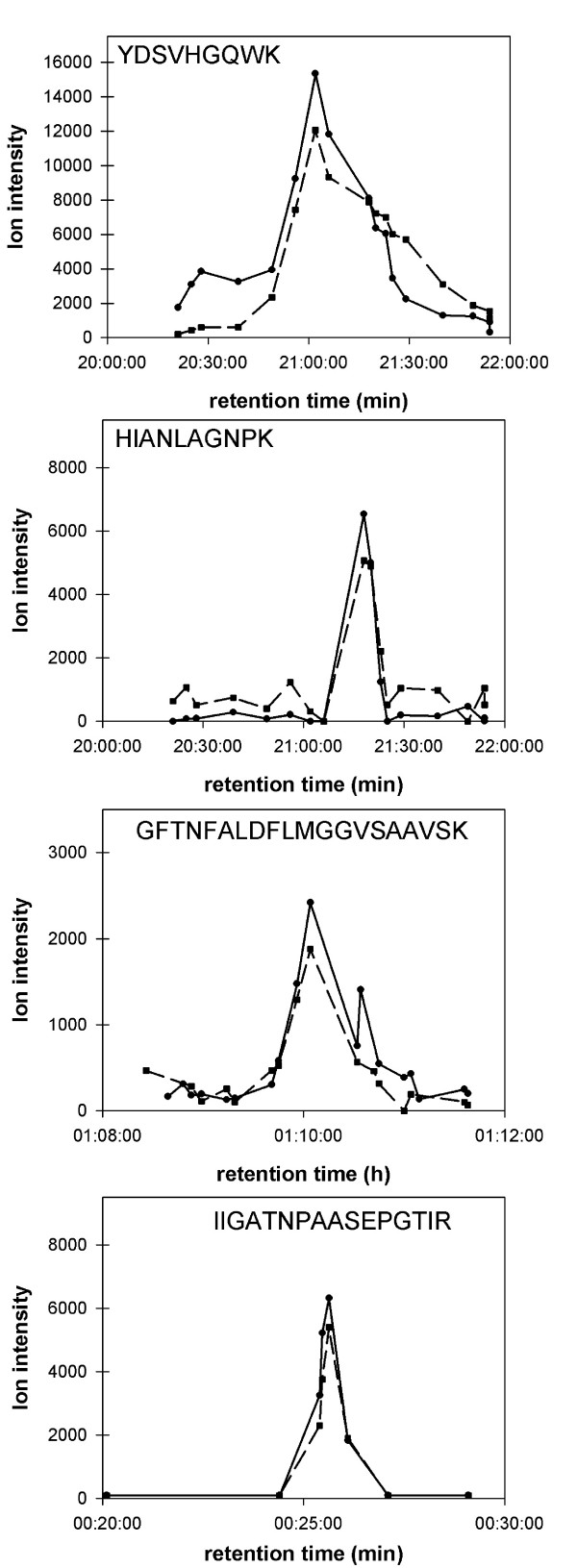
Co-Elution of unlabeled and labeled peptides. Solid lines mark the elution peak of the unlabeled form of each peptide, dashed lines mark the elution peak of the corresponding labeled peptide.

Quantitative Analysis was done using the open-source software MSQuant () which provides a validation and quantitation platform for protein mass spectrometry. The MSQuant software has previously been successfully applied to quantitative analysis involving SILAC labeling [31,32]. For quantitation involving full ^15^N-labeling, ratios between the centroids in the ion chromatograms of the eluting 'heavy' (i.e. ^15^N-labeled) and 'light' (i.e. unlabeled) tryptic peptide peaks were calculated and averaged over the duration of the respective peaks in the total ion chromatogram [26]. Labeled and unlabeled forms of peptides were found to co-elute (Fig. 3) and the quantification was based on the average of independently determined ratios for each peptide. Ratios obtained from different peptides identifying the same protein were averaged. A final standard deviation for protein ratios was calculated from the ratios of the individual peptides or, when the identification was based on a single peptide, from the ratios obtained from the different mass spectra of this peptide pair.

**Figure 4:**
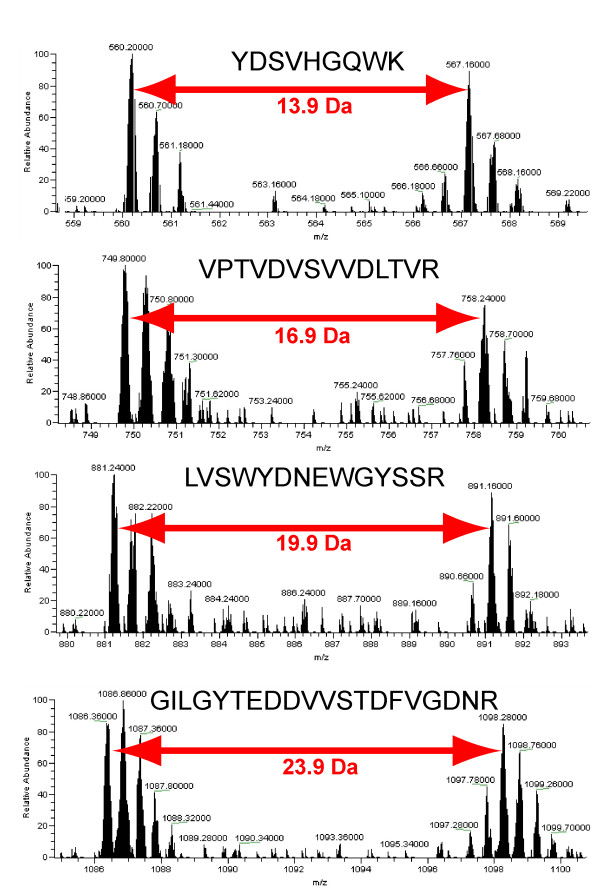
Mass spectra of unlabeled and labeled peptides of from glyceraldehyde-3-phosphate dehydrogenase C subunit (At3g04120) in a 1:1 mixture of unlabeled and labeled protein extracts. For each peptide the mass difference between unlabeled and labeled form of the peptide is different as indicated by the arrows.

The labeled or unlabeled counterparts to peptides identified in the database searches were detected automatically in MSQuant based on their amino acid sequence and mass to charge ratio (Fig. 4). For example, if a peptide was identified in the ^14^N-search, the mass to charge ratio of the corresponding peak in the ^15^N-labeled form was calculated from the expected mass shift based on the amino acid sequence of the ^14^N-isoform of the identified peptide and its charge state.

### Metabolite extraction and sample preparation

Metabolites were extracted from 120 mg cell material, which was shock frozen in liquid nitrogen and ground to a fine powder. Alternately different ratios of labeled and non labeled powder were combined to a final amount of 120 mg and subsequently treated as a single sample. Care was taken to keep samples frozen during manipulation. Frozen material was extracted with 360 μL methanol. Extraction temperature was 70°C. 200 μL chloroform and 400 μL water were added for partitioning polar metabolites as described [54]. Polar extracts were dried in a vacuum concentrator over night. Derivatization was conducted with 10 μL 20 mg mL^-1 ^methoxyamine hydrochloride/pyridine, and 17.5 μL 20 mg mL^-1 ^N-methyl-N-(trimethylsilyl)-trifluoroacetamide. A mixture of alkanes was added for calculation of retention time indices [55].

### Mass spectrometric analysis of metabolites

GC-MS based metabolite profiling performed according to the procedure described previously [56]. Briefly, 1μL of sample was injected at 230°C into a GC 6890 (Agilent Technologies, Palo Alto, CA, USA) gas chromatograph mounted with a Rtx-5Sil MS capillary column, 30 m × 0.25 mm inner diameter with 0.25 μm film thickness, and a 10 m integrated guard column (Restek GmbH, Bad Homburg, Germany). The temperature program comprised 1 min at 70°C, a 9°C min^-1 ^ramp to 350°C, and 5 min at 350°C. The transfer line to the mass-spectrometer was set to 250°C. Carrier gas was helium at a flow rate of 0.6 mL min^-1 ^and operated at constant flow. A time-of-flight mass-spectrometer was used, Pegasus III TOF-MS system (Leco, St. Joseph, MI, USA) with the electron impact ionization source set to -70 eV and 250°C. Data acquisition was set to 20 spectra s^-1 ^and the mass range restricted to m/z = 70–600. All other settings of the mass spectrometer were according to manufacturer's instructions. Chromatograms were acquired, deconvoluted and processed with ChromaTOF™ software (LECO, St. Joseph, MI, USA). Metabolites were identified using the GMD library (Golm Metabolome Database, ; [57,58]). Mass spectral and RI comparison was performed by NIST02 (National Institute of Standards and Technology, Gaithersburg, MD, USA) mass spectral search and comparison software accepting mass spectral matches >650 on a scale of 1000 and retention time index matches ± 5.0 RI units. Mass spectra and fragmentation pattern of amino acid derivatives were interpreted as described [59] in order to identify ^15^N-labeled forms of the respective amino acids.

### Quantitation of metabolites

Absolute ion currents and ratios in ion intensities of metabolites were directly derived from mass spectral fragmentation pattern exported from the ChromaTOF™ software. If available, for each metabolite, more than one fragment ion was used for quantitation [39]. For each sample type (original cell cultures, and the three mixtures), five independent samples were analyzed and the ion intensities for each fragment ion were averaged across the five replica samples.

## Competing interests

The author(s) declare that they have no competing interests.

## Authors' contributions

WRE carried out the experimental work ranging from cell culture labeling to sample preparation and LC-MS/MS analysis, and quantitative evaluation of the protein dataset, and he drafted parts of the manuscript. AE carried out the metabolite mass spectrometric analysis and data evaluation. JK contributed ideas to the metabolomic applications of the labeling strategy and critically reviewed the metabolomic aspects of the manuscript. WXS designed the study and prepared major parts of the manuscript. All authors read and approved the final manuscript.
